# Right- vs. Left-Sided Metastatic Colorectal Cancer: Differences in Tumor Biology and Bevacizumab Efficacy

**DOI:** 10.3390/ijms18061240

**Published:** 2017-06-09

**Authors:** Paola Ulivi, Emanuela Scarpi, Elisa Chiadini, Giorgia Marisi, Martina Valgiusti, Laura Capelli, Andrea Casadei Gardini, Manlio Monti, Silvia Ruscelli, Giovanni Luca Frassineti, Daniele Calistri, Dino Amadori, Alessandro Passardi

**Affiliations:** 1 Biosciences Laboratory, Istituto Scientifico Romagnolo per lo Studio e la Cura dei Tumori (IRST) IRCCS, via P. Maroncelli 40, 47014 Meldola, Italy; paola.ulivi@irst.emr.it (P.U.); chiadini.elisa@irst.emr.it (E.C.); giorgia.marisi@irst.emr.it (G.M.); laura.capelli@irst.emr.it (L.C.); daniele.calistri@irst.emr.it (D.C.); 2 Unit of Biostatistics and Clinical Trials, Istituto Scientifico Romagnolo per lo Studio e la Cura dei Tumori (IRST) IRCCS, via P. Maroncelli 40, 47014 Meldola, Italy; 3 Department of Medical Oncology, Istituto Scientifico Romagnolo per lo Studio e la Cura dei Tumori (IRST) IRCCS, via P. Maroncelli 40, 47014 Meldola, Italy; martina.valgiusti@irst.emr.it (M.V.); andrea.casadei@irst.emr.it (A.C.G.); manlio.monti@irst.emr.it (M.M.); silvia.ruscelli@irst.emr.it (S.R.); giovanni.frassineti@irst.emr.it (G.L.F.); dino.amadori@irst.emr.it (D.A.); alessandro.passardi@irst.emr.it (A.P.)

**Keywords:** metastatic colorectal cancer, bevacizumab, right-sided colon, left-sided colon

## Abstract

There is evidence of a different response to treatment with regard to the primary tumor localization (right-sided or left-sided) in patients with metastatic colorectal cancer (mCRC). We analyzed the different outcomes and biomolecular characteristics in relation to tumor localization in 122 of the 370 patients with metastatic colorectal cancer enrolled onto the phase III prospective multicenter “Italian Trial in Advanced Colorectal Cancer (ITACa)”, randomized to receive first-line chemotherapy (CT) or CT plus bevacizumab (CT + B). *RAS* and *BRAF* mutations; baseline expression levels of circulating vascular endothelial growth factor (VEGF), endothelial nitric oxide synthase (eNOS), cyclooxygenase-2 (COX2), ephrin type-B receptor 4 (EPHB4), hypoxia-inducible factor 1-alpha (HIF-1α), lactate dehydrogenase (LDH), and high-sensitivity C reactive protein (hs-CRP); and inflammatory indexes such as the neutrophil-to-lymphocyte ratio, platelet-lymphocyte rate and systemic immune-inflammation index were evaluated. Patients with right-sided tumors showed a longer median progression-free survival in the CT + B arm than in the CT group (12.6 vs. 9.0 months, respectively, *p* = 0.017). Baseline inflammatory indexes were significantly higher in left-sided tumors, whereas eNOS and EPHB4 expression was significantly higher and *BRAF* mutation more frequent in right-sided tumors. Our data suggest a greater efficacy of the CT + B combination in right-sided mCRC, which might be attributable to the lower inflammatory status and higher expression of pro-angiogenic factors that appear to characterize these tumors.

## 1. Introduction

Colorectal cancer is a heterogeneous disease that can develop in different parts of the colon, with consequent differences in terms of risk factor, histological grade, tumor size and metastatic features [1,2]. Left-sided tumors (originating in the splenic flexure, descending colon, sigmoid colon, rectum, or one-third of the transverse colon) derive from the embryonic hindgut, whereas right-sided tumors (originating in the appendix, cecum, ascending colon, hepatic flexure, or two-thirds of the transverse colon) derive from the embryonic midgut [3].

The different origins consequently lead to tumors with a different gene expression and mutation profile. In particular, right-sided tumors show a higher frequency of *BRAF* mutation and microsatellite instability and more often occur in patients with a genetic predisposition to colorectal cancer (e.g., Lynch syndrome). Conversely, left-sided tumors are characterized by chromosomal instability and a gene expression profile involving the activation of the epidermal growth factor receptor (EGFR) pathway [2,4]. These differences result in different prognoses for the two tumor types, with right-sided tumors associated with poorer patient outcome [2,3,4].

In addition to its prognostic relevance, there is evidence to suggest that tumor localization may be predictive of treatment efficacy with targeted agents, especially those directed against EGFR and vascular endothelial growth factor (VEGF) pathways [3,5,6,7,8,9,10]. Although data on this specific topic are discordant due to the heterogeneity of the studies carried out, left-sided *RAS* wild type (wt) tumors appear to be more responsive to EGFR inhibitors, possibly due to the higher frequency of *BRAF* mutations in right-sided disease [2,7,9,11]. Results on the efficacy of bevacizumab (B) are even more conflicting, some studies finding no correlation with respect to tumor position [12,13] and others, conversely, reporting a link between the effectiveness of the monoclonal antibody and the side of the colon affected [13,14]. Among the latter, some authors found that left-sided or rectal tumors benefited more from B-based treatment [14], whereas others observed that the drug prolonged progression-free (PFS) and overall survival (OS) in right- rather than left-sided tumors [13].

Our extensive research into colorectal cancer revealed a correlation between different biomarkers involved in angiogenic and inflammatory processes and B efficacy [15,16,17,18,19,20], but we have never focused on these different markers in relation to tumor localization. We thus decided to investigate B efficacy, the distribution of a series of parameters involved in angiogenesis and inflammatory processes, and *RAS* and *BRAF* mutations in relation to tumor localization in a case series of metastatic colorectal cancer patients enrolled in the phase III multicenter, prospective, randomized “Italian Trial in Advanced Colorectal Cancer (ITACa)” trial [21] The ITACa trial is registered on ClinicalTrials.gov (NCT01878422).

## 2. Results

### 2.1. Patient Characteristics

The clinical pathological characteristics of patients are reported in Table 1. Sixty patients were randomized to receive chemotherapy (CT) + B and 62 to receive CT alone. In the overall case series 51 and 71 patients had right- and left-sided tumors, respectively. Patient characteristics were well balanced between right- and left-sided tumors. However, a significantly higher percentage of G3 tumors was observed in right-sided disease (*p* = 0.044).

### 2.2. Clinical Outcome in Relation to Tumor Localization

**Figure 1:**
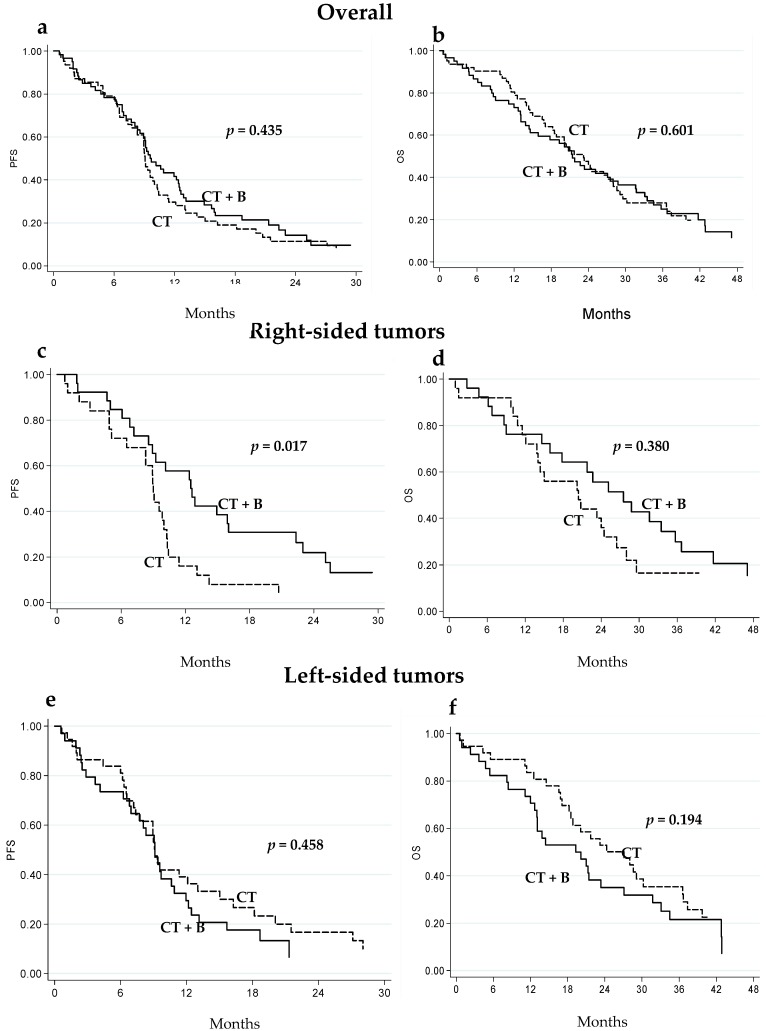
Progression-free (PFS) and overall survival (OS) of mCRC patients treated with CT + B or CT alone: in the overall population (**a**,**b**); in those with right-sided tumors (**c**,**d**); and in those with left-sided tumors (**e**,**f**).

In the overall case series, no significant differences were observed in terms of progression free survival (PFS) or overall survival (OS) in either treatment group (Figure 1a,b and Table 2). Conversely, among right-sided tumors, a better outcome was observed in CT + B patients compared to the CT-only group. In particular, median PFS was 12.6 (95% CI 8.6–16.0) and 9.0 (95% CI 6.5–10.3) months in right-sided CT + B and CT-only patients, respectively (*p* = 0.017) (Figure 1c and Table 2). Significance was maintained after adjusting for CT (FOLFOX4/FOLFIRI), gender, age, ECOG performance status and *KRAS* status (*p* = 0.049). This difference was not observed in patients with left-sided tumors (Figure 1e and Table 2). Similar, albeit not significant, results were observed with regard to median OS: 27.5 (95% CI 15.9–35.7) months for CT + B and 20.4 (95% CI 13.8–26.4) months for CT only in the group with right-sided tumors (*p* = 0.380) (Figure 1d and Table 2). An inverse trend was observed for patients with left-sided tumors (median OS 19.7 (95% confidence interval (CI) 12.7–27.1) and 27.1 (18.2–36.6) months in CT + B and CT-only patients, respectively, *p* = 0.194) (Figure 1f and Table 2). Overall, no differences in terms of PFS or OS were observed between right-sided and left-sided tumors, whereas, within the CT + B group, right-sided tumors were associated with higher, albeit not significant, median PFS and OS (12.6 (8.6–16.0) and 27.5 (15.9–37.7) months, respectively) than left-sided disease (9.1 (6.8–10.9) and 19.7 (12.7–27.1) months, respectively) (*p* = 0.069 and *p* = 0.270, respectively).

### 2.3. Circulating Biomarkers in Right-Aided and Left-Sided Tumors

Overall, higher baseline circulating expression levels of ephrin type-B receptor 4 (EPHB4) and endothelial nitric oxide synthase (eNOS) were observed in right-sided tumors than in left-sided ones. In particular, median relative expression levels of EPHB4 and eNOS were 3.57 (range 0.68–69.55) and 7.14 (range 0.59–123.16), respectively, in right-sided tumors and 2.67 (range 0.21–129.51) and 5.09 (0.41–118.15), respectively, in left-sided lesions (*p* = 0.027 and *p* = 0.036, respectively) (Table 3). No significant differences were observed for the other biomarkers (Table 3).

Significantly lower levels of inflammatory indexes were observed in right-sided compared to left-sided tumors (Table 4). Applying the chosen cut-off for each inflammatory index, neutrophil-to-lymphocyte ratio (NLR) <3 and systemic immune-inflammation index (SII) <730 were more frequently observed in patients with right- vs. left- sided colon cancers (66.7% vs. 44.3%, and 58.8% vs. 40.0%, respectively) (*p* = 0.015 and 0.041, respectively). Platelet-to-lymphocyte ratio (PLR) < 169 was 52.9% and 37.1% in right- and left-sided tumors, respectively (*p* = 0.085). NLR, PLR and SII median values were 2.37 (range 0.90–10.73), 161.76 (range 64.73–310.45) and 641.47 (range 175.42–3614.67), respectively, in right-sided tumors, and 3.19 (range 0.78–12.32), 192.07 (range 38.11–909.72) and 876.77 (range 140.36–8069.24), respectively, in left-sided lesions (*p* = 0.003, *p* = 0.020 and *p* = 0.005, respectively). No differences were seen in high-sensitivity C-reactive protein (hs-CRP) or lactate dehydrogenase (LDH) levels between right- and left-sided tumors (Table 4).

### 2.4. eNOS and VEGF Polymorphism Distribution in Relation to Tumor Localization

No differences were seen in *eNOS* and *VEGF* polymorphism distribution with respect to right- and left-sided tumor localization. With regard to *eNOS* haplotype analysis, the haplotype combination Haplo1/Haplo1 + Haplo2/Haplo2 was found to be more highly represented in right-sided lesions (48.1% vs. 31.0% in left-sided tumors (*p* = 0.050) (data not shown).

### 2.5. RAS and BRAF Mutation Profile

A higher percentage of *BRAF* mutations were observed in right-sided than left-sided tumors (15.7% and 2.8%, respectively) (*p* = 0.017). No significant differences were observed with regard to *KRAS* or *NRAS* mutations in relation to tumor localization (Table 5).

## 3. Discussion

The ITACa trial, a prospective multicenter randomized phase III study whose aim was to evaluate the improvement in PFS obtained by adding B to CT with respect to CT alone, reported similar PFS and OS in the CT and CT + B treatment arms [21]. In the present study, we confirmed the lack of difference in survival between the CT + B and CT-only groups in a subgroup of ITACa patients. Of note, however, a benefit from the addition of B to CT was only seen in right-sided tumors, in agreement with results from the CALGB/SWOG 80405 trial in which treatment with cetuximab led to a clear improvement in PFS and OS in patients with left-sided *KRAS* wt tumors, while B significantly improved survival parameters in those with right-sided lesions [13]. In our study the benefit from B in right-sided tumors was evident for both PFS and OS but was only significant for PFS. However, given that ITACa trial patients who received first-line CT alone went on to receive second-line CT + B, it can be hypothesized that OS may have been impacted by the subsequent lines of therapy.

The distinct molecular features characterizing right- and left-sided tumors account for their different sensitivity to targeted drugs. First, the higher frequency of *BRAF* and *RAS* mutations found in right-sided tumors may explain the higher activity of EGFR inhibitors in left-sided lesions as these alterations represent well known mechanisms of resistance to the drug. However, EGFR inhibitor activity has been shown to be higher in left-sided tumors even when all *RAS* wt patients are considered [3,13], suggesting that other molecular mechanisms such as higher EGFR ligand expression (epiregulin and amphiregulin) might be involved in left-sided lesions [2,22]. The reported higher frequency of *BRAF* mutations in right-sided compared to left-sided tumors, also confirmed in this study, may partially explain the lower responsiveness of right-sided lesions to EGFR inhibitors. Another important factor is histological type. Mucinous cancer is a CRC subtype more frequently found in females and in the right colon. Although this tumor has been associated with poor outcome, its real clinical importance, especially with regard to its response to targeted agents, needs further investigation. There were only three cases of mucinous cancer in our patient population, surprisingly all left-sided. We do not believe this aspect could have influenced clinical results.

Our previous studies focusing on identifying biomarkers predictive of B efficacy indicated that a series of parameters might be associated with different activity of the drug. In particular, we found that specific *eNOS* polymorphisms were correlated with significantly higher PFS and OS in the CT + B group [15]. Interestingly, the present study revealed that *eNOS* polymorphisms associated with higher B efficacy were more frequent in right-sided tumors, reflecting their higher sensitivity to the drug. However, this result was of borderline significance and the biological reason why these polymorphisms were more frequently associated with right-sided tumors remains to be clarified in larger patient population.

We observed a significant difference between right- and left-sided tumors in terms of baseline inflammatory index values. In particular, higher levels of SII, NLR and PLR were found in patients left-sided tumors with respect to right-sided ones. Our previous results showed that, in the overall ITACa case series, patients with low systemic inflammatory indexes (especially low NLR) benefited the most from the addition of B to CT in terms of PFS, suggesting that low systemic inflammatory indexes are associated with an increase in B activity [18]. These results may help to explain the higher activity of B in right-sided tumors in which lower values of SII, NLR and PLR were present. Conversely, we found that circulating levels of biomarkers associated with angiogenesis, i.e., eNOS and EPHB4, were higher in right-sided tumors with respect to left-sided ones, reflecting more marked angiogenesis that may correlate with a greater B efficacy.

It has been demonstrated that tumors with microsatellite instability (MSI), known to be more frequent in right-sided disease, are associated with a higher cytotoxic T-cell infiltration and higher microvessel density, suggesting a higher angiogenic capacity of tumors with this localization [23]. MSI-high CRC with long interspersed nucleotide element-1 (LINE-1) hypomethylation has been seen to have a poor prognosis [24,25], suggesting a complex biological interaction between MSI and LINE-1 hypomethylation. Moreover, differences in the mucosal microbiota of patients who develop right- or left-sided colorectal cancer may also help to explain the different angiogenic and inflammatory properties of the two types of lesions [26]. These findings attest to the better outcome of patients with right-sided tumors treated with B-based treatment.

## 4. Materials and Methods

### 4.1. Case Series

The ITACa protocol was approved by the Local Ethics Committee (Comitato Etico Area Vasta Romagna e I.R.S.T. no. 674) on 19 September 2007, and informed consent was obtained from all patients before blood samples were obtained for genotype testing. Participation in the ITACa biological study was not mandatory for those taking part in the clinical trial. Of the 376 patients with mCRC enrolled onto the ITACa trial, 122 had sufficient archived biological material to be considered for this secondary analysis. Patients were randomized to receive first-line CT (FOLFOX4 or FOLFIRI) only or CT plus B (CT + B). FOLFOX4 consisted of oxaliplatin 85 mg/m^2^ as a two-hour infusion on day 1 and leucovorin 100 mg/m^2^ as a 2-h infusion followed by bolus 5-FU 400 mg/m^2^ and a 22-h infusion of 5-FU 600 mg/m^2^ on days 1–2 every two weeks. FOLFIRI consisted of the same 5-FU + leucovorin regimen with the addition of irinotecan 180 mg/m^2^ as a 90-min infusion on day 1. B was administered as a 30- to 90-min intravenous infusion at a dose of 5 mg/kg on day 1 of each two-week cycle. Treatment was to be continued until progressive disease (PD), withdrawal of consent or unacceptable toxicity, whichever came first. Tumor assessment tests were performed within 28 days of starting the study treatment and repeated every eight weeks during treatment until PD. All patients were evaluated for response (according to Response Evaluation Criteria in Solid Tumors (RECIST) guidelines), PFS and OS.

The study was performed in accordance with the principles of Good Clinical Practice and the ethical standards of the Declaration of Helsinki.

### 4.2. Biomarker Analysis

Laboratory staff blinded to patient outcome performed expression analyses of VEGF-A, cyclooxygenase-2 (COX2), hypoxia inducible factor 1 α (HIF-1α), EPHB4 and eNOS on total RNA extracted from blood collected in PAX-Gene blood RNA kit (PreAnalytix-Qiagen, Hilden, Germany) before the start of therapy, as previously described [20]. hs-CRP and LDH levels were evaluated on serum collected at baseline, as previously reported [16,17].

Information on neutrophil, lymphocyte and platelet counts from blood tests carried out at baseline (before systemic treatment) was collected. SII was calculated as platelet count × neutrophil count/lymphocyte count, NLR was obtained by dividing the absolute neutrophil count by the absolute lymphocyte count, and PLR was calculated by dividing the absolute platelet count by the absolute lymphocyte count [18]. Genotyping analysis of 5 single nucleotide polymorphisms (SNPs) (*VEGF* −2578C>A, −1498C>T, −1154G>A, −634C>G, +936C>T) for *VEGF* and 2 SNPs (*eNOS* −786T>C, +894G>T) and one variable number tandem repeat (VNTR) of 27 nucleotides for *eNOS* was performed on peripheral blood samples, as previously reported [15]. Exons 2, 3 and 4 of *KRAS* and *NRAS* and exon 15 of *BRAF* genes were also analyzed by pyrosequencing, as previously described [27,28].

### 4.3. Statistical Analysis

The objectives of this secondary analysis were to examine the efficacy of B in the ITACa population on the basis of tumor localization and to study the distribution of a series of parameters involved in angiogenesis and in inflammatory processes, together with *RAS* and *BRAF* mutations. The data cut-off for the analysis was 31 December 2013 when the median duration of follow-up was 36 months (range 1–65). The primary aim of the ITACa study was PFS and the secondary endpoint included OS. PFS was defined as the time from random assignment to the first documentation of progressive disease or death from any cause or last tumor evaluation. Patients undergoing curative metastasectomy were censored at the time of surgery. OS was defined as the time interval between random assignment and death or last follow-up visit. PFS and OS were estimated by the Kaplan–Meier method and curves were compared by the logrank test (at a significance level of 5%). Nonparametric tests (Wilcoxon) were used to examine the potential correlation between median baseline circulating biomarker levels and tumor localization.

Receiver Operating Characteristic (ROC) curve analysis was performed to determine the best threshold of hs-PCR levels, with hs-PCR ≥ 13.1 considered as elevated [17]. We distinguished 2 patient subgroups based on LDH levels at baseline: low LDH if within or below the normal range and high LDH if above the upper limit of the normal range [16]. X-tile 3.6.1 software (Yale University, New Haven, CT, USA) was used for bioinformatics analysis of baseline data to determine the cut-off value for pre-treatment levels of each immune-inflammation index. SII ≥ 730, NLR ≥ 3 and PLR ≥ 169 were considered elevated, as previously reported [18]. The association between baseline biomarker levels and tumor localization was evaluated using the Chi-square test. All *p* values were based on two-sided testing. Statistical analyses were performed using SAS statistical software version 9.4 (SAS Inc., Cary, NC, USA).

## 5. Conclusions

Our data suggest a higher benefit of adding B to CT in right-sided mCRC compared to left-sided disease. The efficacy of B may be attributable to a lower systemic inflammatory status and a higher expression of pro-angiogenic factors, both of which appear to characterize patients with right-sided tumors. A prospective validation of these data in a larger patient population is warranted.

## Figures and Tables

**Table 1:** Patient characteristics.

	Total (*n* = 122)	
Patient characteristics	Right-sided (*n* = 51) No. (%)	Left-sided (*n* = 71) No. (%)
Median age, years (range)	68 (37–83)	63 (34–82)
Gender		
Male	28 (54.9)	42 (59.1)
Female	23 (45.1)	29 (40.9)
Performance Status (ECOG)		
0	43 (84.3)	57 (80.3)
1 + 2	8 (15.7)	14 (19.7)
Stage at diagnosis		
I–III	8 (15.7)	20 (28.2)
IV	43 (84.3)	51 (71.8)
Grading		
1 + 2	22 (48.9)	43 (68.2)
3	23 (51.1)	20 (31.7)
Histological type		
Adenocarcinoma NOS	51 (100.0)	68 (95.8)
Mucinous cancer	0	3 (4.2)
CT regimen		
FOLFOX4	31 (60.8)	44 (62.0)
FOLFIRI	20 (39.2)	27 (38.0)
Prior cancer therapy		
Surgery	45 (88.2)	50 (70.4)
Radiotherapy	0	12 (16.9)
Adjuvant CT	10 (19.6)	8 (11.3)
Treatment group		
CT + B	26 (51.0)	34 (47.9)
CT	25 (49.0)	37 (52.1)

CT, chemotherapy; B, bevacizumab; NOS, not otherwise specified.

**Table 2:** PFS and OS in relation to tumor localization in the two treatment groups (CT + B and CT).

		PFS			OS		
	No. Patients	No. Events	Median PFS (months) (95% CI)	*p*	No. Events	Median OS (months) (95% CI)	*p*
Overall							
CT + B	60	53	9.6 (8.3–12.4)		49	21.4 (14.4–28.8)	
CT	62	56	9.1 (8.3–10.0)	0.435	49	23.2 (18.2–28.0)	0.601
Right-sided							
CT + B	26	23	12.6 (8.6–16.0)		21	27.5 (15.9–35.7)	
CT	25	24	9.0 (6.5–10.3)	0.017	20	20.4 (13.8–26.4)	0.380
Left-sided							
CT + B	34	30	9.1 (6.8–10.9)		28	19.7 (12.7–27.1)	
CT	37	32	9.1 (7.2–13.0)	0.458	29	27.1 (18.2–36.6)	0.194

PFS, progression-free survival; OS, overall survival; CT, chemotherapy; B, bevacizumab.

**Table 3:** Median baseline values of circulating biomarkers in relation to tumor localization.

Biomarker	Right-Sided	Left-Sided	*p*
	Median Value (Range)	Median Value (Range)	
VEGF	2.36 (0.68–37.69)	2.22 (0.54–50.80)	0.194
COX	1.37 (0.34–6.07)	1.12 (0.37–4.78)	0.067
HIF1-α	1.17 (0.28–4.23)	1.07 (0.34–5.38)	0.358
EPHB4	3.57 (0.68–69.55)	2.67 (0.21–129.51)	0.027
eNOS	7.14 (0.59–123.16)	5.09 (0.41–118.15)	0.036

**Table 4:** Systemic inflammatory biomarkers in relation to tumor localization.

Biomarker	Total (*n* = 122)		*p*
	Right-Sided (*n* = 51) No. (%)	Left-Sided (*n* = 71) No. (%)	
NLR			
<3	34 (66.7)	31 (44.3)	
≥3	17 (33.3)	39 (55.7)	0.015
PLR			
<169	27 (52.9)	26 (37.1)	
≥169	24 (47.1)	44 (62.9)	0.085
SII			
<730	30 (58.8)	28 (40.0)	
≥730	21 (41.2)	42 (60.0)	0.041
hs-PCR			
<13.1	29 (58.0)	39 (60.0)	
≥13.1	21 (42.0)	26 (40.0)	0.829
LDH			
≤UNL	16 (31.4)	21 (29.6)	
>UNL	35 (68.6)	50 (70.4)	0.832

NLR, neutrophil-to-lymphocyte ratio; PLR, platelet-lymphocyte ratio; SII, systemic immune-inflammation index; hs-PCR, high-sensitivity C-reactive protein; LDH, lactate dehydrogenase; UNL, upper normal limit.

**Table 5:** Gene mutation in relation to tumor localization.

Gene	Total (*n* = 122)		*p*
Patient Characteristics	Right-Sided (*n* = 51) No. (%)	Left-Sided (*n* = 71) No. (%)	
*KRAS*			
Wild type	29 (56.9)	47 (66.2)	0.296
Mutated	22 (43.1)	24 (33.8)	
*BRAF*			
Wild type	43 (84.3)	69 (97.2)	0.017
Mutated	8 (15.7)	2 (2.8)	
*NRAS*			
Wild type	48 (94.1)	70 (98.6)	0.307
Mutated	3 (5.9)	1 (1.4)	

## Abbreviations

mCRC	Metastatic colorectal cancer
CT	Chemotherapy
B	Bevacizumab
VEGF	Vascular endothelial growth factor
eNOS	Endothelial nitric oxide synthase
COX2	Cyclooxygenase-2
EPHB4	Ephrin type-B receptor 4
HIF-1	Hypoxia-inducible factor 1
LDH	Lactate dehydrogenase
hs-CRP	High-sensitivity C-reactive protein
NLR	Neutrophil-lymphocyte ratio
PLR	Platelet-lymphocyte ratio
SII	Systemic immune-inflammation index
PFS	Progression-free survival
OS	Overall survival
